# Compliance with the main preventive measures of COVID‐19 in Spain: The role of knowledge, attitudes, practices, and risk perception

**DOI:** 10.1111/tbed.14364

**Published:** 2021-11-10

**Authors:** María Teresa Beca‐Martínez, María Romay‐Barja, María Falcón‐Romero, Carmen Rodríguez‐Blázquez, Agustín Benito‐Llanes, María João Forjaz

**Affiliations:** ^1^ Servicio de Medicina Preventiva Hospital Virgen de la Salud Toledo Spain; ^2^ Universidad Nacional de Educación a Distancia (UNED) Madrid Spain; ^3^ Centro Nacional de Medicina Tropical Instituto de Salud Carlos III Madrid Spain; ^4^ Universidad de Murcia Murcia Spain; ^5^ Centro Nacional de Epidemiología Instituto de Salud Carlos III Madrid Spain; ^6^ Red de Investigación en Servicios de Salud en Enfermedades Crónicas Madrid Spain

**Keywords:** attitudes, COVID‐19, knowledge, preventive measures, risk perception, Spain

## Abstract

In epidemics such as COVID‐19, major changes need to be made to the population's behavior to prevent infection and stop disease transmission. The three most recommended preventive measures are wearing a mask, washing hands with soap or hydroalcoholic gel, and watching an interpersonal distance of at least two meters (3W) from other people. This study aimed to assess adherence to these COVID‐19‐related three preventive measures and its association with knowledge, attitudes, risk perception, and practices in Spain. The COSMO‐Spain survey, based on the WHO Behavioral Insights questionnaire on COVID‐19, was conducted in the general Spanish population using an online questionnaire (*n* = 1,033). Sociodemographic, knowledge, attitudes, practices, and risk perception variables were included. A multivariable logistic regression model was carried out to evaluate the factors associated with compliance with the three preventive measures. Half of the respondents (49.8%) were women with a median age of 45 (Inter‐quartile Range, IR = 21) years. In the logistic regression, the factors associated with 3W compliance were being over 45 years; knowing about how COVID‐19 spreads and wearing masks properly; appropriate attitudes towards COVID‐19 (greater agreement with mandatory mask use); high risk perception (feeling that the coronavirus is spreading rapidly, being concerned about non‐mask wearers), and adherence to other preventive measures against COVID‐19, such as staying at home. Adequate knowledge, attitudes and risk perception are determinants of 3W compliance. Developing effective health education programs and frequent communication strategies are necessary, particularly for those who adhere less to preventive measures.

## 1 INTRODUCTION

Severe acute respiratory syndrome coronavirus 2 (SARS‐CoV‐2) is the cause of the coronavirus disease 2019 (COVID‐19), a highly contagious disease that spreads rapidly through human‐to‐human transmission (Zhu et al., 2020). The World Health Organization (WHO) declared the COVID‐19 a global pandemic on 1 March 2020 (World Health Organization, 2020).

SARS‐CoV‐2 is transmitted human to human mainly by droplets inhalation in a direct contact with a case, and by indirect contact (airborne and contaminated surfaces or objects) (Lotfi et al., 2020). Human‐to‐human transmission occurs through contact with infected people, through secretions such as infected saliva and respiratory secretions or their respiratory droplets, which are expelled when an infected person coughs, sneezes, talks, or sings when a person is in close contact (within 2 m). Indirect contact can occur if a person touches a surface contaminated with SARS‐CoV‐2, and then hands come into direct contact with mucous membranes such as the eyes, nose, or mouth (WHO, 2020).

In the absence of a treatment and until a higher vaccination rate is reached, the fight against COVID‐19 has been based on behavioural preventive measures. The main individual preventive measures recommended were wearing a mask (regularly), washing your hands (with soap and water for 20 s or with an alcohol‐based lotion) and watching your distance (keeping at least a 2‐m distance from others), such measures being commonly known as the 3W (Centers for Disease Control and Prevention (CDC), 2021). Recently, ventilation of closed places was added (European Centre for Disease Prevention & Controls, 2020). While handwashing has always been one of the key cornerstones of infectious diseases prevention, the use of face masks has recently shown a significant reduction in infections caused by COVID‐19 (Gallaway et al., 2020; Syed et al., 2003; Y. Wang, Ferro, et al., 2020). Also, studies have found that for every meter of distance that is increased, there is a significant reduction in the transmission of SARS‐CoV‐2 (Chu et al., 2020). Other non‐pharmaceutical interventions such as movement restrictions, isolation of people arriving from affected areas or countries, restrictions on mass gathering, quarantine of confirmed cases, close contacts tracing, and testing were also implemented to reduce the spread of COVID‐19 (Ayouni et al., 2021; Davies et al., 2020; Van Scoy et al., 2021; Yu, 2020). Since the COVID‐19 pandemic began, many countries have conducted population behavioural studies to provide scientific evidence to promote prevention (Hosen et al., 2021; Van Nhu et al., 2020). Behavioural insight research is an important tool for understanding behavioural choices, barriers, and drivers within a population (European Centre for Disease Prevention & Control, 2021). Assessment of the adoption of preventive practices against COVID‐19 among population is crucial. Studies about previous outbreaks suggest that knowledge, attitudes, and practices (KAP) survey is the best tool for assessing the population's acceptance and adherence to preventive measures (Ajilore et al., 2017; Person et al., 2004), identifying factors associated with these practices that could help to draw up effective prevention strategies and policies (Chakrawarty et al., 2020). Population behaviour changes depend on the population's knowledge about the disease and individuals’ ability to perceive risks associated with the virus and to adapt their behaviour accordingly (Wise et al., 2020). Previous studies suggested that self‐efficacy in preventing disease and trust in health authorities are also significantly associated with the practice of preventive measures (Chen et al., 2020). Moreover, people´s beliefs about recommended preventive measures and reliance on relevant sources for information about COVID‐19 play an important role in adherence to protective practices (Norman et al., 2020; Ozdemir et al., 2020; Rui et al., 2021; Van Scoy et al., 2021).

Spain left behind its worst COVID‐19 wave and the national lockdown at the beginning of May 2020, with 236,661 cases confirmed and 18,912 deaths (COVID, 2020). At the time of the study, the epidemiological situation improved but there were new outbreaks in several regions and the accumulated incidence was 42.3 cases per 100,000 inhabitants (ISCIII, 2020). Wearing a face mask was compulsory in Spain for all people aged 6 years or older in all open‐air or enclosed public spaces (Boletín Oficial del Estado, 2020). Washing hands and keeping a 2‐m safety distance were recommended. However, little is known about compliance with these three prevention methods and the associated factors.

The study aimed to assess, for first time in Spain, compliance of the 3W preventive measures in the general population and its association with knowledge, attitudes, risk perception, and practices to help understand prevention‐measure adherence and promote the design of evidence‐based information campaigns.

## 2 MATERIALS AND METHODS

### 2.1 Settings and study population

This cross‐sectional study was carried out in Spain. Data were collected between 27 July and 3 August 2020, at the start of a second wave, with the number of national cases rising slowly and new outbreaks in several regions. The use of facemasks was mandatory throughout the country. The survey was conducted by a consumer research company, with a sample matching the Spanish general population in terms of age, education, gender, and area of residence. Methodological aspects have previously been published (Rodríguez‐Blázquez et al., 2021). A nationally representative sample of 1033 people aged 18 and over responded to an online questionnaire on knowledge, attitudes, risk perceptions, preventive practices, and psychological variables related to COVID‐19. The sample was selected through a panel managed by a research company. To maintain the representativeness of the sample, a random stratification by gender, age, geographical area, and educational level was made.

### 2.2 Survey questionnaire

The survey, based on a WHO Behavioural Insights questionnaire and the COVID‐19 Snapshot MOnitoring (COSMO) (Betsch et al., 2020), collected both quantitative and qualitative data to examine citizens' knowledge about the disease and risk perceptions, trust in health authorities and recommendations, adherence to recommended preventive measures, and trust in the sources of information and frequency of consultation.

### 2.3 Variables

An online questionnaire was prepared to collect information on basic socio‐demographic data (sex, age, education level, job status and type, household members, and rural/urban area of residence), COVID‐19 self‐reported infection status, and self‐assessed health, in addition to the main study variables.

Preventive practices were assessed using the question ‘During the last 7 days, which of the following measures have you taken to avoid becoming infected with coronavirus/COVID‐19?’. The listed measures were wearing a facemask according to rules and recommendations, washing my hands often with soap and water, using hydroalcoholic gel or disinfectants for cleaning hands, avoiding public transportation, ensuring physical distancing (at least 2 m), avoiding social/family events, disinfecting surfaces, and staying at home if I have symptoms. Study protocol has been registered within the PsychArchives database (www.psycharchives.org/handle/20.500.12034/4313).

Knowledge about the coronavirus/COVID‐19 was assessed by asking participants about the correctness of 14 statements on coronavirus/COVID‐19 transmission and the correct use of preventive measures. The response options were ‘yes’, ‘no’, and ‘do not know’.

Attitudes towards COVID‐19‐related policies and interventions were assessed by asking about the extent of agreement with decisions taken in Spain for handling the pandemic in general, and with specific measures such as mobility restrictions, compulsory use of facemasks, opening of schools, etc. Answers were rated from 1 (strongly disagree) to 5 (strongly agree).

Trust in institutions was measured by asking ‘How much confidence do you have in (your hospital, health centre, private doctor, company, scientists, WHO, Ministry of Health, regional authorities, public transport, schools, airlines) in addressing the challenges posed by the coronavirus/COVID‐19?’. Answered on a scale from 1 (very low confidence) to 5 (very high confidence).

Risk perception was measured using the following questions: ‘Do you think you could still get coronavirus/COVID‐19?’ (yes, no, or do not know); ‘How severe do you think COVID‐19 disease would be for you if you were infected?’, answered on a scale from 1 (not severe) to 5 (very severe); and the probability of getting infected in different places and activities (public transport, meetings with family and friends, health centres, crowded places, and workplaces), both answered from 1 (very unlikely) to 5 (very likely).

Perceived self‐efficacy was surveyed with the question ‘For me, avoiding be infected with coronavirus/COVID‐19 is…?’ with a response scale from 1 (very difficult) to 5 (very easy).

The population's level of concern about the coronavirus/COVID‐19 outbreak in general and in specific situations was queried using the question ‘At the moment, how much do you worry about…?’, and the answers were rated from 1 (do not worry at all) to 5 (worry a lot). Concerns were measured with the questions ‘I have a feeling that the coronavirus/COVID‐19 is spreading…’ using a scale from 1 (very slow) to 5 (very fast) and ‘Do you believe that the worst of the epidemic…?’ using the answers ‘has passed’, ‘is happening now’, and ‘has not arrived yet’.

Behaviour towards source of information and trust was measured on a scale from 1 (never) to 5 (very often) with the questions ‘How often do you use the following sources to stay informed about COVID‐19?’ and trust in sources of information from 1 (none) to 5 (much) with the question ‘How much do you trust the following sources of information in their reporting about COVID‐19?’.

### 2.4 Data analyses

A descriptive analysis of participants’ characteristics was performed, the categorical variables being described as frequency and percentages with 95% confidence intervals (CIs). The continuous variable was presented using median and inter‐quartile range (IR).

First, a bivariate analysis of the associations between each of the 3W preventive measures with the KAP and risk perception variables was performed. Chi‐squared test was used to study any statistically significant relationship between each of the 3W preventive measures and the KAP or risk perception variables, and *p*‐values <.05 were considered significant.

Multivariable logistic regressions analyses were then performed to assess how COVID‐19‐related factors such as KAP, risk perception, and socio‐demographic characteristics of study participants were associated with adherence to the 3W preventive measures together. The dependent variable was created including only those who answered that they had followed the three preventive measures (wearing facemasks, washing their hands, and ensuring physical distancing) during the last 7 days to avoid becoming infected with COVID‐19. Independent variables were selected through forward stepwise procedure including only those significant at 5% level in a previous chi‐squared test. The odds ratio (OR) and 95% CI were computed, and *p*‐values <.05 were considered statistically significant. All statistical analyses were performed using IBM SPSS Statistics version 27.

## 3 RESULTS

### 3.1 Sample characteristics

The sample was composed of 1033 respondents, 49.8% of whom were women. The mean age was 45.7 (SD: 14.6) years, and 44.9% had attained vocational training, college degree, or higher qualification. The proportion of participants in work was 56.5%. Due to COVID‐19, 5.4% of respondents were unemployed or had been laid off temporarily. Around a third of the sample (33.8%) lived with someone over 60 years old. Most respondents (86.7%) lived in an urban area. Perceived health before COVID‐19 was rated as good/very good by 59.9% participants. A total of 34.2% participants had relatives infected with COVID‐19 and 14.8% had family members who had died from COVID‐19 (Table 1).

**TABLE 1:** Characteristics of the studied respondents (N = 1033)

Characteristic	*n* (%)	95% CI
Sex		
Men	519 (50.2)	47.2–53.3
Women	514 (49.8)	46.7–52.8
Age group, years		
18–29	166 (16.1)	13.8–18.3
30–44	309 (29.9)	27.1–32.7
45–60	344 (33.3)	30.4–36.2
>60	214 (20.7)	18.2–23.2
Education level		
Primary school or below	45 (4.4)	3.1–5.6
Junior high school	206 (19.9)	17.5–22.4
High school	318 (30.8)	28.0–33.6
Vocational training, university degree, or higher	464 (44.9)	41.9–48.0
Job status		
Working	584 (56.5)	53.5–59.5
Unemployed since COVID‐19 or laid off temporarily	56 (5.4)	4.0–6.8
Not working	393 (38.0)	35.1–41.0
Job type		
High risk of contagion	180 (17.4)	15.1–19.7
Moderate contagion risk	344 (33.3)	30.4–36.2
Contagion without risk	270 (26.1)	23.5–28.8
Teleworking	239 (23.1)	20.6–25.7
Household members		
Living with another person between 0 and 13 years old	408 (39.5)	36.5–42.5
Living with another person 14–60 years old	943 (91.3)	89.6–93.0
Living with another person over 60 years old	349 (33.8)	30.9–36.7
Living location		
Rural (<10,000 inhabitants)	137 (13.3)	11.1–15.3
Urban (>10,000 inhabitants)	896 (86.7)	84.7–88.8
Perceived health before COVID‐19		
Good/very good	614 (59.9)	56.4–62.4
Normal	362 (35.0)	32.1–38.0
Bad/very bad	72 (7.0)	5.4–8.5
Family members or relatives infected with COVID‐19		
Yes	353 (34.2)	31.3–37.1
No	680 (65.8)	62.9–68.7
Family members or relatives deceased by COVID‐19		
Yes	153 (14.8)	12.6–17.0
No	880 (85.2)	83.0–87.4

Abbreviation: CI, confidence interval.

### 3.2 Compliance with the 3W preventive measures

In Spain, respondents reported a high level of compliance with each of the 3W preventive measures recommended by the health authorities: 946 (91.6%) respondents said they had followed mask recommendations, 931 (90.1%) said they had washed their hands often, and 875 (84.7%) had kept a physical distance (minimum 2 m) over the last 7 days. Furthermore, most of the population (76.5%) reported having followed the 3W preventive measures together.

### 3.3 Disease knowledge and preventive measures

Implementing the 3W preventive measures at the same time was significantly associated with knowledge of how COVID‐19 spreads: droplets released when coughing/talking, physical contact with someone infected and with contaminated surfaces (*p* <.001). 3W adherence was also associated with knowing that wearing a face mask is useful to avoid infecting others that a mask must cover your nose and mouth (*p* <.001) and other correct face mask usage (see Table 2).

**TABLE 2:** Knowledge about coronavirus disease 2019 (COVID‐19) in Spain and the 3W preventive measures

	Total sample N = 1033	Wearing a mask (*n* = 946)	Washing your hands (*n* = 931)	Watching your distance (*n* = 875)
Knowledge	Yes *n* (%)	Yes *n* (%)	Yes *n* (%)	Yes *n* (%)
COVID‐19 transmission				
Droplets when coughing/talking	974 (94.3)	911 (96.3)*	897 (96.3)*	835 (95.4)*
People who do not have a fever can be contagious	904 (87.5)	834 (89.2)**	822 (88.5)**	772 (88.2)
Physical contact with someone infected	823 (79.7)	772 (81.6)*	758 (81.4)*	723 (82.6)*
Contaminated surface	776 (75.1)	729 (77.1)*	726 (78.0)*	675 (77.1)*
Airborne	613 (59.3)	570 (60.3)**	558 (59.9)	523 (59.8)
Contact with pets (dog, cat, and others)	52 (5.0)	45 (4.8)	47 (5.0)	44 (5.0)
By insect bite	35 (3.4)	28 (3.0)*	29 (3.1)	31 (3.5)
Wearing a mask				
To avoid infecting others	970 (9.3)	906 (95.8)*	894 (96.0)*	838 (95.8)*
It must cover your nose and mouth	889 (86.1)	854 (90.3)*	827 (88.8)*	773 (88.3)*
You must wash your hands before/after use	785 (76.0)	751 (79.4)*	730 (78.4)*	685 (78.3)*
You must only touch the ear tape	736 (71.2)	713 (75.4)*	695 (74.7)*	653 (74.6)*
To protect yourself from being infected	734 (71.1)	685 (72.4)*	671 (72.1)**	641 (73.3)*
Must be removed to cough or sneeze	101 (9.8)	98 (10.4)**	92 (9.9)	90 (10.3)

* Chi‐squared test *p* <.001.

** Chi‐squared test *p* <.050.

### 3.4 Disease attitudes and the 3W preventive measures

Table 3 shows how most people who followed the 3W preventive measures agreed with the mandatory use of masks (*p* <.001). In addition, people who trusted how the epidemic was being managed, mainly by scientists (*p* ≤.050) and health centres (*p* <.001), also complied with the 3W.

**TABLE 3:** Attitudes about coronavirus disease 2019 (COVID‐19) in Spain and the 3W preventive measures

	Total sample N = 1033	Wearing a mask (*n* = 946)	Washing your hands (*n* = 931)	Watching your distance (*n* = 875)
Attitudes	Yes *n* (%)	Yes *n* (%)	Yes *n* (%)	Yes *n* (%)
Agreement with measures to reduce the spread of coronavirus/COVID‐19				
They have been adequate (a)	338 (32.7)	312 (33.0)	301 (32.3)	287 (32.8)
Agreement with the following decisions (a)				
Mandatory use of face masks	822 (79.6)	782 (82.7)*	768 (82.5)*	722 (82.5)*
Autonomous regions draw up regulations	453 (43.9)	411 (43.4)	407 (43.7)	384 (43.9)
Keeping freedom of movement between provinces	393 (38.0)	365 (38.6)	353 (37.9)	329 (37.6)
Opening of schools	351 (34.0)	321 (33.9)	309 (33.2)	294 (33.6)
Keeping freedom of movement between countries	222 (21.5)	193 (20.4)**	188 (20.2)*	181 (20.7)
Confidence in COVID‐19 management (b)				
Scientists	691 (66.9)	655 (69.2)*	641 (68.9)*	600 (68.6)**
My hospital	686 (66.4)	645 (68.2)*	629 (67.6)**	588 (67.2)
My private doctor	615 (59.5)	573 (60.6)**	564 (60.6)**	528 (60.3)
My health centre	600 (58.1)	571 (60.4)*	559 (60.0)*	523 (59.8)*
The Ministry of Health	467 (45.2)	429 (45.3)	429 (46.1)	394 (45.0)
Regional Authorities	424 (41.0)	394 (41.6)	390 (41.9)	367 (41.9)
My company	336 (32.5)	303 (32.0)	305 (32.8)	294 (33.6)
Schools	248 (24.0)	221 (23.4)	220 (23.6)	209 (23.9)
Long distance by trains and buses	164 (15.9)	140 (14.8)*	137 (14.7)*	129 (14.7)*
Public transport	148 (14.3)	129 (13.6)**	126 (13.5)**	121 (13.8)
Airlines	139 (13.5)	111 (11.7)*	113 (12.1)*	110 (12.6)**

* Chi‐squared test *p* <.001.

** Chi‐squared test *p* <.050.

(a) Frequency and percentage of responses ‘agree/completely agree’.

(b) Frequency and percentage of responses ‘much/very much’.

### 3.5 Risk perception, concerns, and the 3W preventive measures

Compliance with the 3W preventive measures was significantly associated with the concern about losing a loved one, overstretched health services, people who did not wear a facemask, and about missing their holidays (*p* <.001) (Table 4). In addition, adherence with the 3W preventive measures was associated with the feeling that COVID‐19 was spreading very fast (*p* <.001) and believing that the worst of the pandemic was not over yet (*p* <.050). Regarding self‐efficacy, thinking that it was easy/very easy to avoid being infected was also associated with compliance with the 3W preventive measures (*p* <.001).

**TABLE 4:** Risk perception and concerns about coronavirus disease 2019 (COVID‐19) in Spain by the 3W preventive measures

	Total sample N = 1033	Wearing a mask (*n* = 946)	Washing your hands (*n* = 931)	Watching your distance (*n* = 875)
Risk perception and concerns	Yes *n* (%)	Yes *n* (%)	Yes *n* (%)	Yes *n* (%)
Do you think you could still be infected with COVID‐19?				
Yes	893 (86.4)	830 (87.7)*	816 (87.6)*	760 (86.9)
How severe do you think the disease would be if you were infected?				
Severe/very severe	448 (43.4)	416 (44.0)	416 (44.7)**	391 (44.7)**
Probabilities of infection with COVID‐19 at (a)				
Crowded places	864 (83.6)	815 (86.2)*	799 (85.8)*	748 (85.5)*
Public transport	679 (65.7)	643 (67.1)*	625 (67.1)**	586 (67.0)
Health centre	600 (58.1)	555 (58.7)	548 (58.9)**	516 (59.0)
Meeting with family/friends at home	524 (50.7)	486 (52.4)	482 (51.8)	458 (52.3)**
Working outside	495 (47.9)	451 (47.7)	449 (48.2)	417 (47.7)
Shopping	341 (33.0)	303 (32.0)**	301 (32.3)	283 (32.3)
How much do you worry about COVID‐19 (b)	925 (89.5)	607 (64.2)	609 (65.4)*	575 (65.7)
Regarding COVID‐19 I am concerned about (b)				
Losing a loved one	873 (84.5)	820 (86.7)*	815 (87.5)*	752 (85.9)*
Overstretched health services	838 (81.8)	787 (83.2)*	779 (83.7)*	728 (83.2)*
People who do not wear a mask	827 (80.1)	779 (82.3)*	770 (82.7)*	723 (82.6)*
A new lockdown	754 (73.0)	710 (75.1)*	699 (75.1)*	641 (73.3)
My physical and mental health	600 (58.1)	557 (58.9)	556 (59.7)*	506 (57.8)
Going out into the street	360 (34.8)	319 (33.7)*	326 (35.0)	302 (34.5)
Not being able to pay my bills	543 (52.6)	500 (52.9)	498 (53.5)	458 (52.3)
Closure of nurseries and schools	532 (51.5)	485 (51.3)	489 (52.5)**	446 (51.0)
Work‐life balance	488 (47.2)	447 (47.3)	439 (47.2)	417 (47.7)
Missing my holidays	233 (21.6)	184 (19.5)*	187 (20.1)*	176 (20.1)*
I feel that COVID‐19 is spreading				
Fast/very fast	741 (71.7)	695 (73.5)*	684 (73.5)*	643 (73.5)*
Do you believe the worst of the pandemic				
Is not over yet	438 (42.4)	417 (44.1)*	406 (43.6)**	385 (44.0)**
Has passed	358 (34.7)	329 (34.8)	323 (34.7)	291 (33.3)**
Is happening now	237 (22.9)	200 (21.1)*	202 (21.7)*	199 (22.7)
As things are currently, avoiding be infected with coronavirus/COVID‐19 is				
Easy/very easy	318 (30.8)	279 (29.5)*	275 (29.5)*	263 (30.1)*

* Chi‐squared test *p* <.001.

** Chi‐squared test *p* <.050.

(a) Frequency and percentage of responses ‘likely/very likely’.

(b) Frequency and percentage of responses ‘much/very much’.

### 3.6 Sources and confidence in the information

**FIGURE 1:**
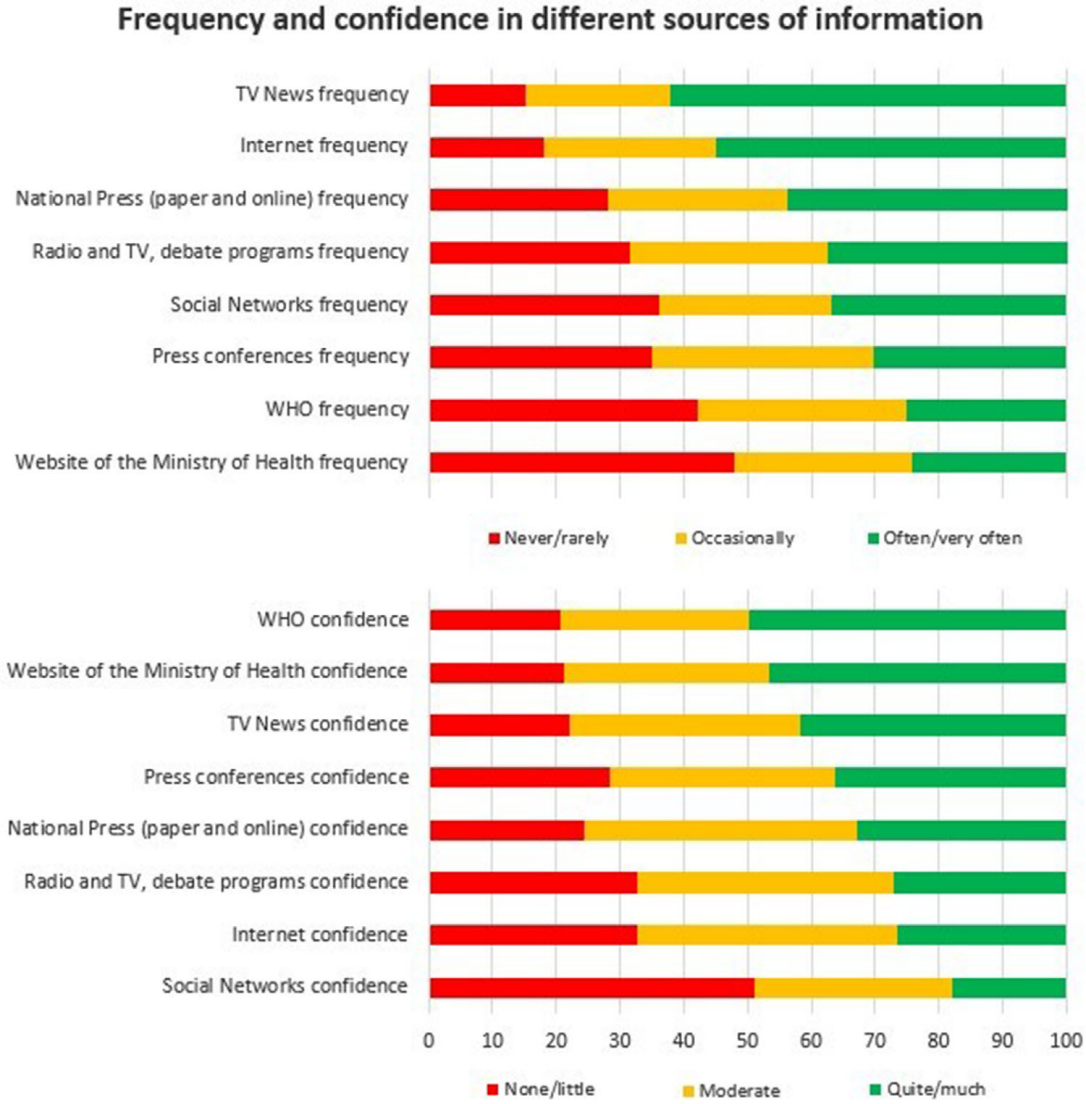
Frequency and confidence in different sources of information about coronavirus/coronavirus disease 2019 (COVID‐19)

Most of respondents were very confident in the information provided by their health care professionals (717, 69.4%), and this confidence was significantly associated with the implementation of the 3W preventive measures. Figure 1 shows the frequency and confidence in other sources of information. Checking TV news frequently was significantly associated (*p* <.001) with following each of the 3W preventive measures, while checking the Spanish Ministry of Health's website was significantly associated with washing hands 92.5% (*p* = .017) and watching distance 87% (*p* = .048). Trust in the information delivered by social media (e.g. Facebook, Twitter, YouTube, and WhatsApp) was also associated with adherence to the 3W (*p* < .001).

### 3.7 Preventive practices

Implementing one of the 3W preventive measures was significantly associated with implementing either of the other two. In addition to high compliance with the 3W preventive measures, 86% of the interviewees said they had used hydroalcoholic gel and 64% had avoided using public transport. Also, 57% of the participants had avoided meeting relatives or friends, and 55% had disinfected surfaces to avoid COVID‐19 infection (see Table 5).

**TABLE 5:** Practices to avoid coronavirus disease 2019 (COVID‐19) infection in Spain and the 3W preventive measures

	Total (N = 1033)	Wearing a mask (*n* = 946)	Washing your hands (*n* = 931)	Watching your distance (*n* = 875)
Practices	Yes *n* (%)	Yes *n* (%)	Yes *n* (%)	Yes *n* (%)
Using a mask following recommendations	946 (91.6)	**–**	885 (95.1)*	826 (94.4)*
Washing my hands often	931 (90.1)	885 (93.6)*	**–**	819 (93.6)*
Using hydroalcoholic gel	894 (86.5)	817 (86.4)	809 (86.9)	762 (87.1)
Watching your physical distance (2 m minimum)	875 (84.7)	826 (87.3)*	819 (88.0)*	**–**
Avoiding public transport	663 (64.2)	607 (64.2)	599 (64.3)	556 (63.5)
Not going to social/family meetings	587 (56.8)	544 (57.5)	526 (56.5)	497 (56.8)
Disinfecting surfaces (doorknobs, doorbell…)	569 (55.1)	519 (54.9)	507 (54.5)	479 (54.7)
Staying at home if I have symptoms	337 (32.6)	308 (32.6)	306 (32.9)	277 (31.7)
Not leaving the house/leaving it as little as possible/telecommute	132 (12.8)	131 (13.8)*	131 (14.1)*	132 (15.1)*

* Chi‐squared test *p* <.001.

### 3.8 Factors associated with adherence to all the 3W preventive measures regarding COVID‐19

In the multiple logistic regression analysis, knowing at least one form of transmission (OR = 2.46) that masks are used to avoid infecting others (OR = 2.38) and their correct use (OR = 1.58) was significantly associated with adherence to the 3W preventive measures. Age was the only socio‐demographic characteristic associated with adherence to the 3W measures (OR = 1.76).

Moreover, those who agreed with mandatory use of masks and who were worried about ‘people who do not wear a mask’ were almost twice as likely to comply with the 3W preventive measures. Feeling that coronavirus/COVID‐19 was spreading fast was also associated with the 3W preventive measures adherence.

By comparison, people who did not trust in long‐distance trains and buses had 1.60 probability to follow the 3W preventive measures. Finally, people who trusted the coronavirus/COVID‐19 information given by healthcare professionals were almost twice as likely to comply with the 3W preventive measures, while people who trusted social media (e.g. Facebook, Twitter, YouTube, and WhatsApp) were 0.60 times likely to adhere to them (Table 6).

**TABLE 6:** 3W preventive measure determinants

	Adjusted OR (95% CI)
Age	
45–60	1.76 (1.19–2.58)**
>60	2.27 (1.37–3.78)*
Most common forms of COVID‐19 contagion	
Droplets when coughing/talking	2.46 (1.30–4.66)**
Wearing a mask	
To avoid infecting others	2.38 (1.23–4.59)**
You must wash your hands before and after use	1.65 (1.10–2.46)**
You must only touch the ear tape	1.58 (1.06–2.23)**
Agreement with the following decisions (a)	
Mandatory use of facemasks	1.77 (1.18–2.67)**
Confidence in this institution to address COVID‐19 (b)	
Confidence in long‐distance trains and buses	0.60 (0.39–0.93)**
Risk perception	
I have a feeling that coronavirus/COVID‐19 is spreading fast	1.59 (1.11–2.26)**
Regarding COVID‐19 I am concerned about	
People who do not wear a mask	1.81 (1.21–2.70)**
Confidence in coronavirus/COVID‐19 information from (b)	
Healthcare professionals	1.75 (1.22–2.53)**
Social Media (e.g. Facebook, Twitter, YouTube, and WhatsApp)	0.60 (0.39–0.92)**
Practices	
Not leaving the house/leaving as little as possible/teleworking	39.56 (5.43–288.28)*

* Chi‐squared test *p* <.001.

** Chi‐squared test *p* <.050.

(a) Agree/completely agree.

(b) Much/very much.

## 4 DISCUSSION

The adoption of behavioural preventive measures against COVID‐19 has been critical in controlling the pandemic. According to our study, adherence to the main preventive measures was associated with good knowledge about COVID‐19 transmission; positive attitudes about mandatory use of face masks, with having some concerns about the pandemic and how fast COVID‐19 was spreading, and with trust in specialized information sources. In terms of practices, compliance with one of the studied preventive measures was associated with adherence to the other two, and all the 3W were associated with trying to leave the house as little as possible (MacIntyre et al., 2021).

In Spain, compliance with the 3W main preventive measures (wearing a mask, washing your hands, and watching your distance) was associated with age. People over 45 years old followed the preventive measures more closely than the younger population. Similar results have been found in countries such as the United Kingdom, Greece, and the United States, where younger adults reported lower rates of full compliance with recommended behaviours (Lennon et al., 2020; MacIntyre et al., 2021; Mouchtouri et al., 2020; Norman et al., 2020), and in line with findings from previous pandemics (Chen et al., 2020). Knowledge is a prerequisite for forming positive attitudes and promoting positive behaviours in high transmissible pandemics such as COVID‐19 (Norman et al., 2020). Several studies stated how good knowledge in the community about COVID‐19 multiplied adherence to preventive practices against the disease, such as wearing a face mask, washing your hands with soap and water regularly, using a personal alcohol‐based sanitizer, avoiding crowded areas and closed spaces, or not travelling out of state (Hatabu et al., 2020; Khasawneh et al., 2020; Nwonwu et al., 2020). In Spain, compliance with the all three preventive measures was associated with knowing the most mentioned COVID‐19 modes of transmission and how to wear a mask properly. These variables were also associated with greater compliance in other studies (Ferdous et al., 2020; Mouchtouri et al., 2020).

Attitudes such as agreeing with the mandatory use of masks in Spain, and being worried about people not wearing them also determined adherence to the 3W preventive measures, as observed in other studies (Cartaud et al., 2020; Gray et al., 2020; X. Wang, Tian, et al., 2020). Awareness of face masks use is more present than other recommended practices of infection control for breaking the COVID‐19 transmission chain (Beesoon et al., 2020). In a study conducted in different English‐speaking countries, almost all participants believed that when wearing a mask, they also needed to wash their hands and adhere to physical distancing (MacIntyre et al., 2021). Public health messages should emphasize the combination of the 3W instead of focusing on only one of them such as facemasks that can produce a false sense of protection.

Risk perceptions also influence individuals’ protective behaviour. Complying with the 3W preventive measures was associated with the feeling that coronavirus/COVID‐19 was spreading fast, as well as with leaving the house as little as possible and telework. Risk perception was high in the population. Even so, at the time of the study, Spain had a relatively low accumulated incidence (ISCIII, 2020). Previous studies have shown similar results about how people´s concerns and risk‐perceptions contributed to preventive practices (Banerjee et al., 2021; Mouchtouri et al., 2020; Nazione et al., 2021; Ozdemir et al., 2020; Qian et al., 2020; Rui et al., 2021; Van Scoy et al., 2021), suggesting that evaluating these risk perceptions is critical for an effective and appropriate crisis response (Ajilore et al., 2017). On the contrary, preventive behaviour, including hand‐washing and social distancing, decreased in people less concerned about COVID‐19 or perceiving the risk to be exaggerated or disproportionate to the threat (Kantor & Kantor, 2020; Ozdemir et al., 2020).

Individuals with accurate health information are usually more motivated to engage in preventive health behaviour (Li et al., 2020; Qian et al., 2020). However, the volume of rumours about COVID‐19 makes individuals vulnerable to misinformation (Cuan‐Baltazar et al., 2020). One factor that could potentially affect information credibility is its source. In Spain, people who were more confident about coronavirus/COVID‐19 information from healthcare professionals were more likely to adhere to the 3W preventive measures in line with studies where reliance on expert sources encouraged protective behaviour (Ali et al., 2020; Rui et al., 2021; Sakya et al., 2021), while people who had trusted social media (e.g. Facebook, Twitter, YouTube, and WhatsApp) adhered much less to the 3W preventive measures. This could be explained by the false and misleading information that was spread over the Internet and different types of social media platforms, despite the efforts to limit its dissemination (Anwar et al., 2020; Cuan‐Baltazar et al., 2020). Internet searches for ‘face mask’ have risen since February 2020, and this could indicate not only an increase of face mask awareness but might also reflect proactively engaging in wearing a mask as a preventive measure (Lin et al., 2020). The WHO highlighted the need to promote the use of official public health organizations’ websites when seeking information on COVID‐19 preventive measures (Hernández‐García & Giménez‐Júlvez, 2020). The Internet and social media play an important role in circulating information, influence public behaviour, and can help to prevent the disease, but could also have negative impacts if they are not effectively used in outbreaks (Anwar et al., 2020; Cuan‐Baltazar et al., 2020). Appropriate educational and risk communication strategies could foster engagement in protective behaviours.

Mass public health educational campaigns are essential to inform and update the population about the COVID‐19 pandemic and to address rumours and misinformation, which may hamper efforts to fight the virus (Mboya et al., 2020). Local‐level coordination of contextually appropriate strategies is likely to be the cornerstone of the national response (World Health Organization, 2020). Coordination of regional and district/council stakeholders such as the regional and district commissioners, local health management teams, religious leaders, political leaders, healthcare workers, the media, and non‐governmental organizations is critical to improve the implementation of and compliance with preventive strategies (Betsch et al., 2020). Coordinated efforts among stakeholders in the country are necessary to contain COVID‐19 through context‐specific prevention strategies. Such interventions should also focus on ensuring compliance with the national and international recommended preventive measures. However, due to the evolving nature of this pandemic, it is clear that messaging needs to be dynamic and continuously updated in line with evidence gathered and changing recommendations (Mboya et al., 2020).

There is a complex interplay of changes in epidemiology data, media attention, pandemic control measures, risk perception, and public health behaviour (Reintjes et al., 2016). An effective risk communication in a pandemic such as COVID‐19 must include information not just about the threat itself but also about how people perceive and respond to that threat (Abrams & Greenhawt, 2020; Varghese et al., 2021). Data gathered in our study could support effective interaction among authorities, health workers, journalists, and the public to design effective risk communication that encourages appropriate behavioural change, helps manage the crisis, and protects the most important asset in a crisis: public trust (Betsch et al., 2020).

The main limitation of this study is that it is based on an online‐based survey, thereby restricted to only those with Internet access. However, the sample matched the Spanish general population in terms of age, education, sex, and area of residence. This is a cross‐sectional study, and as the COVID‐19 situation is changing rapidly, the results reported here represent the situation during the surveyed period.

### 4.1 Conclusions and recommendations

Our study provides information that could help authorities to monitor and improve adherence to the preventive measures in force. Reinforcing the population's knowledge, positive attitudes, adequate practices, and risk‐perception during epidemics such as COVID‐19 is crucial to controlling the disease. Authorities have to develop effective, evidence‐based health education programmes and risk communication campaigns to promote a change in behaviours aimed primarily at those who adhere less to recommended preventive measures. According to our study, it would be particularly important to target the subset of individuals who remain disengaged like young people, with low self‐efficacy and low risk perception and who do not look for information early on the pandemic. Public health messages should emphasize the combination of the 3W, instead of focusing on only one of them such as facemasks and would reach more credibility if healthcare professionals transmit these messages. However, messaging needs to be dynamic and continuously updated in line with scientific evidence gathered and changing recommendations.

## CONFLICT OF INTEREST

The authors declare no conflict of interest.

## ETHICS STATEMENT

The authors confirm that the ethical policies of the journal, as noted on the journal's author guidelines page, have been adhered to. This study was approved by Ethics Committee of the Spanish National Health Institute, Carlos III (CEI PI 59_2020‐v2).

## AUTHOR CONTRIBUTIONS

María Romay‐Barja, María Falcón‐Romero, Carmen Rodríguez‐Blázquez, and María João Forjaz conceived the study. María Teresa Beca‐Martínez and María Romay‐Barja analyzed the data. María Teresa Beca‐Martínez and María Romay‐Barja wrote the initial draft of the manuscript. María João Forjaz was involved in funding acquisition and project administration. All authors collaborated in writing, review, and editing the manuscript.

## Data Availability

All necessary data are included in the manuscript and can be obtained from the authors upon request.
